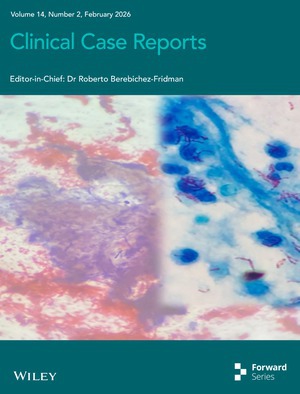# Cover Image

**DOI:** 10.1002/ccr3.72211

**Published:** 2026-03-05

**Authors:** Anupriya Sah, Iccha Kumar Maharjan, Pragya Regmee, Abhinaya Luitel

## Abstract

The cover image is based on the article *A Granulomatous Puzzle: Tubercular Lymphadenitis Without AFB Clues* by Anupriya Sah et al., https://doi.org/10.1002/ccr3.71997.